# Attitudes and experiences towards the IMMUNI App among Sapienza university students: a pilot study

**DOI:** 10.1093/eurpub/ckac129.419

**Published:** 2022-10-25

**Authors:** MR De Blasiis, C Isonne, F Turatto, E Mazzalai, C Marzuillo, C De Vito, P Villari, V Baccolini

**Affiliations:** Public Health and Infectious Disease, Sapienza University, Rome, Italy

## Abstract

**Background:**

IMMUNI is an app that was created to help fight epidemics, starting with COVID-19. The app has a contact tracing feature but its diffusion in Italy was low. In this pilot study, we investigated university students’ attitudes and experience towards the IMMUNI app.

**Methods:**

This cross-sectional study was conducted at Sapienza University of Rome between 14 April and 19 April 2021. An online survey was administrated to university students of medical area. A multivariable logistic regression model was built to identify app download's predictors. Adjusted odds ratio (aOR) and 95% confidence interval (CI) were calculated.

**Results:**

We collected 247 questionnaires (response rate: 78.2%). More than half of the students (65.0%) didn't download IMMUNI app mostly because of the belief that it was useless (30.0%). By contrast, the main reason for downloading was sense of duty (40.0%). Experience with the app was limited but the process was judged as lacking for the technical difficulties. As for hypothetical incentives, feedback on how the download could help against the pandemic was considered as the most effective (3.5 out of 5). In the multivariable analysis, higher likelihood of download was associated with higher fear of contagion for family and acquaintances (aOR:1.50, 95% CI: 1.01-2.23) and higher rating to the health management of the emergency (aOR: 1.33, 95% CI:1.00-1.76). The highest odds of download were found for participants who have been advised to download the app (aOR: 3.21, 95% CI:1.80-5.73). On the other hand, greater belief that the virus came from a laboratory was negatively associated with the download (aOR: 0.75, 95% CI: 0.60-0.93).

**Conclusions:**

Strategies aimed at raising students’ awareness on the importance of health technologies, restoring confidence in health authorities, and limiting disinformation around SARS-Cov-2 should be devised. In addition, the app could be enriched with positive feedbacks for users, and some technical issues should be fixed.

**Key messages:**

• The spread of digital technologies for public health purposes is fostered by trust in health institutions and improving health literacy and user engagement.

• An efficient and user-friendly technology is recommended.

